# Psychosocial Hazard Analysis in a Heterogeneous Workforce: Determinants of Work Stress in Blue- and White-Collar Workers of the European Steel Industry

**DOI:** 10.3389/fpubh.2017.00210

**Published:** 2017-08-17

**Authors:** Yannick Arnold Metzler, Silja Bellingrath

**Affiliations:** ^1^Work and Organizational Psychology, University of Duisburg-Essen, Essen, Germany

**Keywords:** workload, Copenhagen Psychosocial Questionnaire, effort–reward imbalance, occupational stress, steel industry

## Abstract

The European steel industry’s workforce is highly heterogeneous and consists of various occupational groups, presumably facing different psychosocial stressors. The few existing studies on the subject mainly focused on physical constraints of blue-collar workers, whereas the supposable psychosocial workload received only little research attention. This is remarkable considering the challenges associated with statutory required risk assessment of psychosocial hazards. Valid measures of hazard analysis must account for various stressors and reliably identify them, also between occupational groups. The present study, based on a sample of blue- and white-collar workers (*N* = 124) from the European steel industry, aims to provide a first insight into psychosocial stressors and strain at work in this rarely researched industrial sector. Furthermore, two well-known theoretical roadmaps in job analysis are examined regarding their utility for risk assessment in heterogeneous workforces: the German standard version of the Copenhagen Psychosocial Questionnaire (COPSOQ) and the short version of the effort–reward imbalance questionnaire. Hierarchical multiple regression analyses revealed that the COPSOQ was better suited to predict various strain indices in the present sample. Especially stressors relating to socioemotional aspects, such as work-privacy conflict, revealed a reasonable impact, indicating the need for comprehensive solutions at the organizational level instead of solutions focusing on single workplaces. To conclude, a broadly diversified and validated approach in psychosocial risk assessment is needed to adequately assess the variety of psychosocial factors at work and in different occupational groups.

## Introduction

Sector-specific characteristics of psychosocial stressors and strain in the European steel industry have rarely been investigated so far. Considering the fact that this branch is one of the key industries in Europe, as well as in Germany, this is surprising. Existing sources describe several potential work specific stressors mainly caused by heavy work, organizational factors, and economic conditions (1). Based on the fifth European Working Conditions Survey, the European Foundation for the Improvement of Living and Working Conditions (2) concludes that employees working in the metal industry are generally exposed to high physical risks, atypical working hours in terms of shift work, high demands, and low autonomy. In line with these findings, the occupational medical French SUMER survey of the DARES (French Ministry of Labour) emphasizes for example constraints in posture and articular stress, noise pollution, and manual handling of loads as main physical demands (3). The Stress Report of the German Federal Institute of Occupational Safety and Health (BAuA) notes that employees within the metal industry report reoccurring work processes, predefined standards in quantity, performance and time, low influence and a detailed prescription of workmanship. In addition, a good cooperation with colleagues and being part of a community are mentioned as resources (4). These conditions already illustrate a variety of presumable sources possibly evoking psychosocial stress, especially when keeping in mind that job demands, control and social interactions are known to be potent predictors of adverse work-related outcomes (5–9).

Besides the above mentioned aspects, restructuring and downsizing of the steel industry due to a challenging globalized market lead to consequences like job losses, perceived job insecurity and various changes in work activities and organizations (1, 10), resulting in increasing levels of psychosocial stress (11). Even though the steel industry is primarily a male blue-collar working sector (10), the need for indispensable business operating areas such as sales and distribution, research and development or financial administration result in a heterogeneous workforce (1), including also white-collar occupations that underlie equal sector-specific circumstances. Traditionally, there has been a greater interest in adverse outcomes caused by environmental and physical factors, and thus a major focus on examining blue-collar steel workers. Besides general sources describing such types of occupational hazards as published from the International Iron and Steel Institute and the United Nations Environment Programme (12) as well as the International Labor Organization (13), there has been a number of studies investigating the impact of heavy work on for instance low back pain in different steel working occupations (14–16). Furthermore, a large number of studies on exposure to chemical and other physical hazards across different industries have been published, which are not further dealt with here. Less, however, is known about the characteristics and structure of possible psychosocial stressors in the European steel industry’s work environment. Büssing (17) for example assessed the psychological consequences of job insecurity in the German steel industry. Further research emphasis has been placed on the analysis of shift work such as the impact of differing shift systems on sleep quality or cardiovascular risks (18, 19), or the significance of work experience in limiting fatigue and preventing accidents during night shifts (20). Whereas the studies summarized above addressed the relationship between a specific, predefined risk factor and various strain outcomes, the aim of the present study is a more general evaluation of potential stressor—strain associations in the German steel industry. It is not clear whether the steel industry’s work environment and the resulting determinants of strain differ from or, respectively correspond to previous findings in other industrial sectors. This is important since regulations and procedures on psychosocial risk assessment formulated by institutions of the European Union, e.g., the framework directive 89/391/EEC (21) and the guidance on risk assessment at work (22), demand employers to measure, evaluate, and deal with psychosocial hazards at work. To conclude, we hypothesize the steel industry’s workforce to be a rather heterogeneous and highly risk-exposed occupational group that may face various presumable sources of psychosocial stress. Hence, we consider that a universal, wide-ranged measurement approach is needed to adequately examine and compare both psychosocial workload and strain in this setting.

Several instruments assessing psychosocial factors at work have been developed in the past years (23, 24), usually aiming to quantify perceived workload by employees on a Likert scale-based rating procedure. Besides known and validated instruments of stress and job analysis developed in Germany like the Trier Inventory for Chronic Stress (25), the salutogenic subjective work analysis (SALSA) (26), or the instrument for stress-oriented job analysis (ISTA) (27), a remarkable number of instruments for psychosocial hazard analysis in Germany consists of short forms, checklists, and/or non-validated questionnaires. Such approaches seem questionable, since neither sound evidence nor the diverse facets of workplace characteristics become visible. Validated and comprehensive measurement approaches should be preferred in this respect. Scientific literature and practice often refer to the effort–reward imbalance (ERI) model (6) as a theoretical roadmap for describing the emergence and characteristics of psychosocial stress. During the last decade it has proven its predictive ability in a large number of prospective epidemiological cohort studies, including different working populations as well as in experimental setups, especially with regard to cardiovascular diseases and affective disorders, but also with regard to further negative health outcomes (28–31). The ERI model postulates psychosocial work stress to be a result of failed social reciprocity, thereby emphasizing social factors in the etiology of health and disease. Siegrist (6) hypothesizes a recurrent disappointment in the basic principle of reciprocity at work, namely an imbalance between high efforts spent and low rewards received, to affect health and well-being by compromising crucial self-regulatory functions and eliciting negative emotions and associated psychobiological stress responses (6, 32). The ERI model furthermore states that failed social reciprocity is often experienced by individuals with little or no alternative choices in the labor market, those exposed to a strong job competition and those characterized by higher levels of overcommitment. This intrinsic component, a motivational pattern of excessive work-related commitment is assumed to strengthen the perception of failed social reciprocity but was also shown to independently affect health outcomes (33–35). With respect to the stress-theoretical basis of the model, the experience of ERI is assumed to result in a sustained activation of the stress axes and to trigger distinct areas in the brain’s reward circuitry, thereby suppressing the production of dopamine and oxytocin, neurotransmitters with stress-buffering properties. In the setting of industrial occupations, Schmidt et al. (36) for instance examined the role of ERI as a predictor of the metabolic syndrome in a German cohort study of industrial employees (*N* = 4.141). The analysis showed that employees belonging to the high ERI group had a 29% higher chance for developing a metabolic syndrome compared to the low ERI group. A further study based on data of an occupational cohort (*N* = 2.674) by Li et al. (37) revealed high ERI to be related to diabetes and prediabetes with a 27% chance greater compared to low ERI in men.

Another approach to assess the determinants of psychosocial stress at work, which is not based on one specific theoretical construct, is the Copenhagen Psychosocial Questionnaire (COPSOQ) (38) of the Danish National Institute for Occupational Health. Aiming to assess psychosocial workload on a broader level, several of the established theories concerning psychosocial stress at work were combined: (1) the job characteristics model, (2) the Michigan organizational stress model, (3) the demand–control–(support) model, (4) the socio-technical approach, (5) the action–theoretical approach, (6) the ERI model, and (7) the vitamin model (39, 40). The elucidation of these models is beyond the scope of this article. The COPSOQ does not substantially consist of newly developed items; instead, its scales are mainly composed of already existing and validated instruments, like, for instance, the Setterlind Stress Profile (41) or the Short-Form 36 health survey (42). Thus, the COPSOQ represents a multifactorial approach to assess a variety of constructs in order to establish a combined risk profile. The aim to achieve high construct validity regarding psychosocial factors at work in general (43) makes it a valuable instrument especially for operational practices (44) such as psychosocial risk assessment. The COPSOQ, furthermore, includes items assessing stressors such as quantitative demands, influence at work or quality of leadership as well as strain indices such as job satisfaction, or self-reported general health. The COPSOQ scales have been widely used in Danish and international studies (43, 45–47). A prospective study examining psychosocial workload in terms of the COPSOQ as a risk factor for long-term sickness absence among 5.141 Danish employees (46), for example, concluded that emotional demands and demands for hiding emotions significantly predicted long-term sickness absence among men. Another prospective study from Denmark (*N* = 4.133) measured the impact of psychosocial work characteristics on the incidence of severe depressive symptoms (47). The analysis revealed an increased risk for women with low influence at work and low support from supervisors, whereas, men were at severe risk when they considered their jobs to be precarious. Recent reviews furthermore report associations of psychosocial factors at work with musculoskeletal disorders (48–50), mental health problems, mostly depressive symptoms and anxiety (7, 51, 52), and organizational outcomes such as job satisfaction (53, 54) or accidents and injuries, as was recently demonstrated in a sample of Iranian steel workers (55). Cooper et al. (56) suggest workplace stress to be responsible for 60–80% of all work-related accidents.

### Study Objectives

The present data were collected as part of a pilot study to examine psychosocial hazards in terms of occupational risk assessment in a German company of the steel industry. The objectives of this study were (1) to provide insight into the possibly wide-ranged scope of psychosocial work stressors and strain in the European steel industry and (2) to attain information about the utility of the COPOSQ and the ERI-questionnaire for psychosocial risk assessment, especially in view of the steel industry’s heterogenous workforce. (2.1) The variance explained by belonging to different working groups (blue collar and white collar) was examined, as a sufficient discriminatory power can be informative for determining plausible differences and information about relevant psychosocial factors between occupational groups. This knowledge is crucial since measures of hazard analysis must provide Occupational Health and Safety (OHS) with clear information about workplace characteristics. (2.2) We, then, tested both the COPSOQ- and the ERI-questionnaire in view of their own as well as their combined power in predicting work-related outcomes measured by the COPSOQ.

## Materials and Methods

### Study Sample

The current study is based on cross-sectional data from a sample of 124 employees working in the same German steel-manufacturing company. The sample includes two working areas, an administrative (*N* = 74, white-collar occupation) and a production area (*N* = 50, blue-collar occupation). Next to sociodemographic characteristics (gender and age in groups), hierarchical position, working volume, length of company affiliation, length of working experience, and type of working contract were measured as additional confounders. The white-collar group mainly consisted of sales representatives in strategy and planning, while the blue-collar group comprised tasks, such as welding, scaffold construction, and metal maintenance works, organized in fully continuous shift systems.

### Procedure

The study took place in March 2016 and was approved by the occupational review boards for data protection and work safety. A working councils’ agreement including the data protection officer’s agreement ensuring compliance with all relevant privacy policy regulations according to German law was adopted. A member of the working council was present during the data collection process to ensure compliance with the agreement and to guarantee anonymity and voluntary participation. Completed questionnaires had to be put into a sealed ballot box by the employees. To increase acceptance and response rate, the required time to complete the questionnaires was granted as working time. Furthermore, the employees were surveyed directly at the workplace, were supplied with information material before and could ask questions during the survey. A full response rate was acquired.

### Measures

#### Effort–Reward-Imbalance Questionnaire

To assess ERI, we used its validated German short form, consisting of the three 4-point Likert-scaled dimensions effort (3 items), reward (7 items), and overcommitment (6 items). All scales are calculated as sum scores. Effort scores range from 3 to 12, reward from 7 to 28, and overcommitment from 6 to 24, pointing two low or high efforts spent, rewards received and overcommitment, respectively. The imbalance between effort and reward is constructed as a ratio with the effort score in the nominator and the reward score in the denominator. To adjust the unequal number of items, the ratio is multiplied with a correction factor calculated as the ratio of reward and effort items (57).

#### Copenhagen Psychosocial Questionnaire

The standard version of the German COPSOQ (44) examines psychosocial workload and strain by the four main scales: demands (14 items, subscales quantitative demands, emotional demands, demands for hiding emotions, work-privacy conflict), influence and development (19 items, subscales influence at work, degree of freedom, possibilities for development, meaning of work, workplace commitment), interpersonal relations and leadership (26 items, subscales predictability, role-clarity, role-conflicts, quality of leadership, social support, feedback, social relations, sense of community, mobbing), outcomes (24 items, subscales intention to leave the job, job satisfaction, general health, personal burnout, cognitive stress symptoms, satisfaction with life), and one further scale on insecurity at work (4 items). These dimensions consist of 25 subscales, which we extended by the subscale trust and justice (4 items, interpersonal relations and leadership) with reference to the Freiburg research centre for occupational sciences (FFAW) (58). Item responses score on Likert scales of 4, 5, and 7 points on a 0–100 range. All scales are calculated as average scores. In order to facilitate the interpretation of our results, the coding of the COPSOQ scales was slightly modified in the present study. Higher average scores now universally indicate higher psychosocial stressors and strain.

### Statistical Analysis

Concerning the COPSOQ, less than 1% of all values contained missing data. Little’s MCAR test (*p* = 0.525) and graphical analysis showed no evidence for a systematic bias. We, therefore, imputed missing values of the COPSOQ by the mean score of the respective subscale, if at least half of its items were rated. Otherwise, the rating was declared as missing. In view of the ERI, we considered list-wise deletion to be more appropriate because most of its missing data occurred from only three almost unanswered questionnaires. In general, the application of advanced techniques, such as multiple imputation, would have been a disproportional effort to generate just a small amount of data. In a first step, we compared the levels of stress and strain assessed with the COPSOQ with available reference data on a descriptive level (59). Afterward, Cronbach’s alpha was assessed to provide reliability measures. To determine how belonging to the blue- or white-collar working group may affect the ratings, we calculated partial eta^2^ as effect size measure for all subscales using analysis of variance (ANOVA). Partial eta^2^ reflects the extent to which proportions of variance are attributable to a categorical variable and is similar to *R*^2^ in multiple linear regression (60). As our sample is non-representative, we compared our results with those of the German COPSOQ validation study. Cohen (61) suggests explained variances from 1% to indicate a small, from 6% a medium and from 14% a large effect. We, therefore, only present explained variances of partial eta^2^ ≥ 0.01. To assess the ability of the ERI and the COPSOQ to predict the strain outcomes inherent in the COPSOQ, we conducted hierarchical multiple regression analyses for each questionnaire. We also tested whether the predictive power would increase by combining both models.

## Results

### Sample Characteristics

Table 1 shows the sample characteristics and distributions of the following covariates: number of participants, age in groups, gender, hierarchical position, working volume, average length of company affiliation in years, average length of working experience in years (current activity) and type of working contract, each per working group and in total. Obtainable information showed that 88 (71%) participants were male and 32 (28.5%) female. Age was categorized in eight groups for reasons of data protection. The median of 4.5 indicates most participants to be of middle to older age. The total average length of company affiliation was 22 years and the total average length of working experience 14 years. The table reveals clear differences of the socio-demographic characteristics between the two groups. The blue-collar group for instance solely consists of older male workers whereas the white-collar group almost equally comprises younger to middle-aged men and women. This, however, depicts the industry’s prevailing structure as has been mentioned in the Section “Introduction.”

**Table 1:** Sample characteristics.

Working group	Blue collar	White collar	Total
*N*	50	74	124
Median grouped age	6	4	4.5
Gender M/W	49/0	39/32	88/32
Position (employee/specialist/supervisor)	41/8/1	55/1/15	96/9/16
Working volume (full time/>50% time/<50% time)	50/0/0	67/4/3	117/4/3
Average length of company affiliation in years	27	19	22
Average length of working experience in years	17	12	14
Type of working contract (fixed-term yes/no)	6/43	7/67	13/110

*M, men, W, women*.

*Age groups from 1 to 8: (1) up to 25 years, (2) 26–30 years, (3) 31–35 years, (4) 36–40 years, (5) 41–45 years, (6) 46–50 years, (7) 51–55 years, (8) over 55 years*.

**Figure 1:**
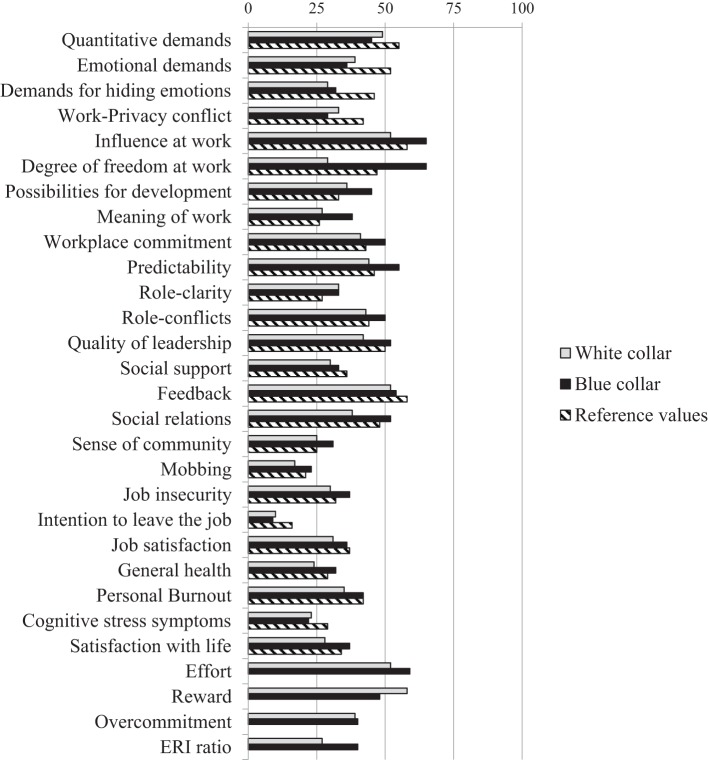
Average ratings of the scales compared to available reference values (59). No references are available for figures of effort–reward imbalance (ERI). Coding of the references was adjusted to our computation (0, stressor not present, 100, maximum presence of stressor).

Figure 1 presents the average scores of the COPSOQ scales within both working groups, compared to the reference values available from the German COPSOQ-databank (*N* = 10.022) (59). This databank does not encompass a population-based representative sample; however, it provides a job-exposure matrix mostly reflecting the actual distribution of occupations in Germany. In the context of hazard evaluation, scorings higher than the reference value indicate a need for action. In Germany, classifying psychosocial hazards as risks requires either (1) using standardized instructions provided by the research tool, (2) comparing own data with available reference values for detecting discrepancies, (3) elaborating and evaluating hazards via group discussions, or (4) conducting strain analyses (62). Examining the figures reveals that especially the ratings of the blue-collar group mostly exceed the reference values. Scores are especially high on scales like predictability (lack of information), role-conflicts, social relations, and the entire influence dimension and exceed both the reference values and the average ratings of the white-collar group. The white-collar group only shows slight deviations from the reference values in the scales possibilities for development and meaning of work and a stronger one in role-clarity. To facilitate the comparability, we transposed the ERI scales and the ERI-ratio to a 0–100 range in accordance to the COPSOQ scales (see Figure 1). In this case, no global reference values were available. Yet, Nübling et al. (43) provide comparative figures for several different occupations.

### Reliability of the Scales and Variance Explained by Working Group

All in all, the scales show satisfying reliability scores in terms of Cronbach’s alpha (see Table 2). Especially when keeping in mind that most scales used in the study are comprised of only 4–8 items and a higher number of items is likely to result in higher Cronbach’s alpha values (63). Merely, the scales feedback (α = 0.49) and social relations (α = 0.27) revealed low reliability scores (both 2-item scales) and were, therefore, not included in the analyses.

**Table 2:** Cronbach’s alpha of the scales.

Scales	Number of items	Cronbach’s α
**Copenhagen Psychosocial Questionnaire**		
Quantitative demands	4	0.75
Emotional demands	3	0.80
Demands for hiding emotions	2	0.81
Work-privacy conflict	5	0.92
Influence at work	4	0.79
Degree of freedom at work	4	0.81
Possibilities for development	4	0.84
Meaning of work	3	0.86
Workplace commitment	4	0.77
Predictability	2	0.67
Role-clarity	4	0.76
Role-conflicts	4	0.73
Quality of leadership	4	0.93
Social support	4	0.72
Sense of community	3	0.83
Mobbing	1	–
Trust and justice	4	0.82
Job insecurity	4	0.65
Intention to leave the job	1	–
Job satisfaction	7	0.82
General health	1	–
Personal Burnout	6	0.86
Cognitive stress symptoms	4	0.84
Satisfaction with life	5	0.89
**Effort–Reward Imbalance (ERI)**		
Effort	3	0.63
Reward	7	0.75
Overcommitment	6	0.79
Effort–reward (ERI) ratio	Interaction term	–

As described above, effect size measures were then computed to examine the power of both instruments in distinguishing different types of psychosocial stressors and strain (see Table 3). As expected, partial eta^2^ estimates differed between the present study and the German COPSOQ validation study, although some similar patterns became apparent. The scale degree of freedom at work showed the highest amounts of variance explained by working group in both our $\left(\eta_{p}^{2}=0.53\right)$ and the validation study $\left(\eta_{p}^{2}=0.47\right)$. With respect to the scales degree of freedom at work [M(blue collar) = 65, M(white collar) = 29] and influence at work [M(blue collar) = 65, M(white collar) = 52], the effect size measures indicated lower (perceived) degrees of freedom and influence at work within the industrial occupations group. Similar conclusions can be drawn with respect to the other scales. In view of the ERI, the reward scale and the ERI-ratio revealed significant medium effects corresponding to the descriptive results.

**Table 3:** Analysis of variance to assess explained variance by working group (blue collar versus white collar) compared to the German validation study.

Scales	Current study		Validation study
	*F*	$\eta_{p}^{2}$	$\eta_{p}^{2}$
**Copenhagen Psychosocial Questionnaire**			
Quantitative demands	1.875 (1, 117)	0.02	0.12**
Emotional demands	0.521 (1, 117)	0.00	0.25**
Demands for hiding emotions	0.637 (1, 119)	0.01	0.03**
Work-privacy conflict	0.558 (1, 120)	0.01	0.16**
Influence at work	15.237 (1, 118)	0.11**	0.07**
Degree of freedom at work	132.972 (1, 117)	0.53**	0.47**
Possibilities for development	6.937 (1, 121)	0.05*	0.13**
Meaning of work	9.796 (1, 121)	0.08**	0.12**
Workplace commitment	5.645 (1, 121)	0.05*	0.07**
Predictability	9.703 (1, 121)	0.07**	0.05**
Role-clarity	0.015 (1, 119)	0.00	0.05**
Role-conflicts	4.178 (1, 120)	0.03*	0.04**
Quality of leadership	4.504 (1, 120)	0.04*	0.04**
Social support	0.844 (1, 121)	0.01	0.06**
Sense of community	3.956 (1, 122)	0.03*	0.02**
Mobbing	2.485 (1, 121)	0.02	0.03**
Trust and justice	2.999 (1, 118)	0.03	–
Job insecurity	3.307 (1, 122)	0.03	0.10**
Intention to leave the job	0.263 (1, 120)	0.00	0.02**
Job satisfaction	4.841 (1, 118)	0.04*	0.02**
General health	5.723 (1, 121)	0.05*	0.05**
Personal Burnout	7.452 (1, 122)	0.06**	0.11**
Cognitive stress symptoms	0.791 (1, 122)	0.00	0.02**
Satisfaction with life	8.615 (1, 121)	0.07**	0.02**
**Effort–Reward Imbalance (ERI)**			
Effort	3.742 (1, 115)	0.03	–
Reward	10.929 (1, 1,009)	0.09**	–
Overcommitment	0.107 (1, 114)	0.00	–
ERI-ratio	13.474 (1, 107)	0.11**	–

*Validation study [*N* = 2,561, Nübling et al. (44)] did not include ERI*.

*p < 0.05.

**p < 0.01.

### Regression Models

In a next step, we examined which stressors and psychosocial workplace characteristics in terms of the COPSOQ and ERI scales predict self-reported strain assessed with the COPSOQ. Considering the substantial number of potential predictors, hierarchical multiple regression analyses were carried out to achieve a better accuracy. Tables 4–9 present first the COPSOQ’s regression analyses for each outcome. While model 1 always presents the covariates only, predictors were then included in stages if they improved the model by explaining at least an additional increase of two percent of variance (*R*^2^). If not, they were excluded. We accepted these minor changes in *R*^2^ to be relevant. When keeping the multifactorial stress–strain relation in mind it is likely that desirable associations are already found at low to moderate levels (44, 64). Thus, the following models show the accepted predictors and their standardized beta weight, as well as *R*^2^, adjusted *R*^2^ and change in *R*^2^.

**Table 4:** Hierarchical regression analysis of predictors of intention to leave the job (Copenhagen Psychosocial Questionnaire model).

Predictors	Model 1	Model 2	Model 3
	β	β	β
Working area	−0.002	−0.096	−0.105
Gender	−0.094	−0.149	−0.159
Age	−0.215	−0.273	−0.324
Position	0.109	0.170	0.196
Working volume	0.271	0.196	0.145
Company affiliation	0.010	0.032	0.059
Working experience	−0.060	−0.031	−0.040
Type of working contract	−0.253*	−0.196	−0.115
Meaning of work		0.337**	0.293**
Social support			0.248*
*R*^2^	0.094	0.188*	0.238**
adj. *R*^2^	0.015	0.107*	0.153**
Δ *R*^2^	0.094	0.094**	0.050*

*Predictors were included if they explained at least additional 2% of variance*.

*p < 0.05.

**p < 0.01.

***p < 0.001.

**Table 5:** Hierarchical regression analysis of predictors of job satisfaction (Copenhagen Psychosocial Questionnaire model).

Predictors	Model 1	Model 2	Model 3	Model 4	Model 5
	β	β	β	β	β
Working area	0.327**	−0.027	−0.065	−0.149	−0.141
Gender	0.140	0.153	0.059	0.126	0.116
Age	−0.159	−0.289	−0.317*	−0.378**	−0.356**
Position	−0.263*	−0.190*	−0.113	−0.097	−0.080
Working volume	0.245	0.178	0.078	0.038	0.021
Company affiliation	0.033	0.076	0.082	0.177	0.166
Working experience	0.073	−0.007	0.056	0.122	0.118
Type of working contract	−0.184	−0.138	−0.073	−0.005	0.015
Degree of freedom at work		0.540***	0.348**	0.357***	0.339***
Meaning of work			0.550***	0.398***	0.350***
Predictability				0.350***	0.221**
Quality of leadership					0.257**
*R*^2^	0.275**	0.396**	0.627**	0.700**	0.737**
adj. *R*^2^	0.207**	0.332**	0.582**	0.659**	0.698**
Δ *R*^2^	0.275**	0.121**	0.231**	0.072**	0.037**

*Predictors were included if they explained at least additional 2% of variance*.

*p < 0.05.

**p < 0.01.

***p < 0.001.

**Table 6:** Hierarchical regression analysis of predictors of general health (Copenhagen Psychosocial Questionnaire model).

Predictors	Model 1	Model 2	Model 3
	β	β	β
Working area	0.100	0.097	0.125
Gender	0.178	0.178	0.151
Age	0.406*	0.412*	0.408*
Position	−0.200	−0.158	−0.115
Working volume	−0.058	−0.051	−0.014
Company affiliation	−0.215	−0.241	−0.238
Working experience	0.188	0.209	0.141
Type of working contract	0.182	0.154	0.075
Quantitative demands		−0.106	−0.294*
Work-privacy conflict			0.332**
*R*^2^	0.189*	0.198*	0.271**
adj. *R*^2^	0.117*	0.116*	0.187**
Δ *R*^2^	0.189*	0.021	0.052**

*Predictors were included if they explained at least additional 2% of variance*.

*p < 0.05.

**p < 0.01.

***p < 0.001.

**Table 7:** Hierarchical regression analysis of predictors of personal burnout (Copenhagen Psychosocial Questionnaire model).

Predictors	Model 1	Model 2	Model 3	Model 4
	β	β	β	β
Working area	0.324**	0.349**	0.368***	0.288**
Gender	0.228	0.156	0.169	0.122
Age	−0.134	−0.250	−0.229	−0.315*
Position	0.060	−0.002	−0.002	0.041
Working volume	0.028	0.080	0.079	0.027
Company affiliation	−0.078	−0.042	−0.007	0.084
Working experience	0.077	0.014	−0.037	−0.055
Type of working contract	0.076	0.096	0.061	0.149
Emotional demands		0.407***	0.248**	0.246*
Work-privacy conflict			0.302**	0.263**
Sense of community				0.312**
*R*^2^	0.154*	0.288***	0.352***	0.433***
adj. *R*^2^	0.080*	0.217***	0.279***	0.363***
Δ *R*^2^	0.154*	0.134***	0.064**	0.081**

*Predictors were included if they explained at least additional 2% of variance*.

*p < 0.05.

**p < 0.01.

***p < 0.001.

**Table 8:** Hierarchical regression analysis of predictors of cognitive stress symptoms (Copenhagen Psychosocial Questionnaire model).

Predictors	Model 1	Model 2	Model 3
	β	β	β
Working area	0.029	0.054	0.010
Gender	0.233	0.219	0.193
Age	−0.177	−0.194	−0.239
Position	−0.044	−0.067	−0.043
Working volume	0.145	0.163	0.135
Company affiliation	0.046	0.093	0.139
Working experience	0.061	−0.032	−0.044
Type of working contract	−0.017	−0.042	0.006
Work-privacy conflict		0.306**	0.284**
Sense of community			0.170
*R*^2^	0.139	0.224**	0.248**
adj. *R*^2^	0.065	0.149**	0.166**
Δ *R*^2^	0.139	0.085**	0.024

*Predictors were included if they explained at least additional 2% of variance*.

*p < 0.05.

**p < 0.01.

***p < 0.001.

**Table 9:** Hierarchical regression analysis of predictors of satisfaction with life (Copenhagen Psychosocial Questionnaire model).

Predictors	Model 1	Model 2
	β	β
Working area	0.206*	0.124
Gender	−0.026	−0.034
Age	0.180	0.163
Position	−0.236*	−0.094
Working volume	0.478***	0.335**
Company affiliation	−0.143	−0.136
Working experience	0.068	0.073
Type of working contract	−0.029	0.022
Possibilities for development		0.391***
*R*^2^	0.297***	0.405***
adj. *R*^2^	0.237***	0.346***
Δ *R*^2^	0.297***	0.108***

*Predictors were included if they explained at least additional 2% of variance*.

*p < 0.05.

**p < 0.01.

***p < 0.001.

Job satisfaction was the outcome with the highest amount of variance explained. The most frequently observed significant predictor was work-privacy conflict, associated with general health, personal burnout, and cognitive stress symptoms. Furthermore, especially scales like meaning of work, social support and work-privacy conflict predicted several of the strain indices. Almost all subscales conformed to theory considering the (linear) relationship between stress and strain. Merely, the negative relationship between quantitative demands and general health is arguable, as it does not fit to common theory at first glance. Since both working groups scored rather low on this scale [M(blue collar) = 32, M(white collar) = 24], it is conceivable that an exposure at this level is perceived positively. A greater range of scorings in quantitative demands might have resulted in different findings. Surprisingly, the scale job insecurity showed no significant contribution at all. The ERI-questionnaire offers the possibility to assess the outcomes on a subscale level or via the ratio. Regression models of the transposed subscales effort, reward, and overcommitment, are shown in Tables 10–13. Although an association between the scales and the strain indices can be determined, the predictive power was significantly lower compared to the COPSOQ-models. The outcomes intention to leave the job and general health had to be excluded because of violations to the model fit. Job satisfaction again showed the largest amount of variance explained and the scales overcommitment and reward were the most frequent predictors. Testing the ratio (results not shown) revealed a further substantial loss in predictive power, as the ratio merely explained 9% of variance in job satisfaction (*R*^2^ = 0.088, *p* < 0.001) above the covariates. We also assessed whether combining both instruments would increase the predictive power. As this approach was not accompanied by significant improvements, we decided not to present these results.

**Table 10:** Hierarchical regression analysis of predictors of job satisfaction (effort–reward imbalance model).

Predictors	Model 1	Model 2
	β	β
Working area	0.282*	0.094
Gender	0.106	0.114
Age	−0.164	−0.229
Position	−0.261*	−0.107
Working volume	0.287*	0.100
Company affiliation	0.058	0.068
Working experience	0.091	0.124
Type of working contract	−0.240*	−0.223*
Reward		−0.566***
*R*^2^	0.259**	0.49***
adj. *R*^2^	0.187**	0.433***
Δ *R*^2^	0.259**	0.231***

*Predictors were included if they explained at least additional 2% of variance*.

*p < 0.05.

**p < 0.01.

***p < 0.001.

**Table 11:** Hierarchical regression analysis of predictors of personal burnout (effort–reward imbalance model).

Predictors	Model 1	Model 2	Model 3
	β	β	β
Working area	0.350**	0.261*	0.238*
Gender	0.161	0.156	0.148
Age	−0.168	−0.064	−0.125
Position	0.093	−0.003	−0.024
Working volume	0.090	0.045	0.010
Company affiliation	−0.120	−0.083	−0.019
Working experience	0.131	0.075	0.074
Type of working contract	0.059	0.159	0.155
Effort		0.322**	0.231**
Overcommitment			0.271**
*R*^2^	0.186*	0.271**	0.332***
adj. *R*^2^	0.112*	0.195**	0.255***
Δ *R*^2^	0.186*	0.084**	0.062**

*Predictors were included if they explained at least additional 2% of variance*.

*p < 0.05.

**p < 0.01.

***p < 0.001.

**Table 12:** Hierarchical regression analysis of predictors of cognitive stress symptoms (effort–reward imbalance model).

Predictors	Model 1	Model 2	Model 3
	β	β	β
Working area	0.038	−0.025	−0.050
Gender	0.188	0.185	0.176
Age	−0.159	−0.086	−0.155
Position	−0.022	−0.089	−0.113
Working volume	0.185	0.154	0.114
Company affiliation	0.006	0.031	0.104
Working experience	0.029	−0.011	−0.012
Type of working contract	−0.022	0.048	0.044
Effort		0.226	0.124
Overcommitment			0.306
*R*^2^	0.138	0.180*	0.258**
adj. *R*^2^	0.060	0.095*	0.172**
Δ *R*^2^	0.138	0.042*	0.079**

*Predictors were included if they explained at least additional 2% of variance*.

*p < 0.05.

**p < 0.01.

***p < 0.001.

**Table 13:** Hierarchical regression analysis of predictors of life satisfaction (effort–reward imbalance model).

Predictors	Model 1	Model 2
	β	β
Working area	0.210*	0.108
Gender	−0.076	−0.072
Age	0.137	0.100
Position	−0.217*	−0.133
Working volume	0.562***	0.460**
Company affiliation	−0.138	−0.132
Working experience	0.063	0.084
Type of working contract	−0.063	−0.054
Reward		−0.310**
*R*^2^	0.326***	0.395***
adj. *R*^2^	0.260***	0.328***
Δ *R*^2^	0.326***	0.069**

*Predictors were included if they explained at least additional 2% of variance*.

*p < 0.05.

**p < 0.01.

***p < 0.001.

## Discussion

In the current study, stressor–strain relationships were assessed in a sample of 124 blue- and white-collar employees of a German steel-manufacturing company, using the German standard version of the COPSOQ and the short version of the ERI-questionnaire. Conducted in the scope of psychosocial risk assessment, we wanted to give a first insight into possible psychosocial stressors and associated strain outcomes in this rarely researched industrial sector and to assess the usefulness of both measures for hazard analysis in a heterogenous workforce. As principal results, the ANOVA showed that belonging to the white- or blue-collar working group explained greater variations in the COPOSQ’s scales than in the scales of ERI. Since measures of hazard analysis must reliably distinguish stressors between occupational groups, the wide-ranged measurement approach of the COPSOQ appears suitable for this purpose. Considering the stress–strain relation, hierarchical multiple regression analysis revealed that both the COPSOQ and the ERI explained distinct proportions of variance above the covariates. However, the explanatory power of the COPSOQ was significantly stronger with respect to all outcome variables measured. The results of our analysis are discussed below, especially considering conclusions for occupational risk assessment and a comparison between the two questionnaires if appropriate.

We determined a satisfactory discrimination between blue- and white-collar workers in the COPSOQ’s scales influence at work and degree of freedom at work and significant medium effects with respect to the ERI-ratio and the reward scale. The COPSOQ scales better distinguished between working groups than the ERI-questionnaire, most likely due to its broader measurement approach. Medium to large effects were observed with respect to influence at work and degree of freedom, indicating reasonable and plausible differences between the blue- and the white-collar group. Expected differences in work-privacy conflict could not be detected. In general, work-life conflicts are usually investigated in white-collar occupations whereas it is of equal importance in blue-collar occupations (65). However, some factors assessed by the COPSOQ (e.g., meaning of work, quality of leadership, commitment) are of a general scope and may hardly provide information about specific groups. Concerning the ERI scales, both the reward scale and the ratio distinguished between working groups with medium effect sizes. As the reward scale also assesses aspects of satisfaction (“Considering all my efforts and achievements, my salary/income is adequate”), this scale differs considerably from others, such as degree of freedom at work, which rather characterizes the work activity as such. Congruent reflections may apply in view of the ratio as a type of a general self-rated evaluation parameter of work in contrary to a descriptive rating of work characteristics. Thus, since reference values are not available for the ERI-questionnaire and as the characteristics that underlie the reward scale and the ratio are of a more general nature, inferences regarding hazard analysis must be drawn with caution. We finally conclude that both instruments can reliably distinguish distinct types of psychosocial work stress in different occupational groups, albeit the variety of the COPSOQ scales allows a much broader and more precise analysis, indicating its worth for hazard analysis.

With respect to associations between stressors and strain outcomes, hierarchical multiple regression analysis revealed that the COPSOQ had a stronger explanatory power than the ERI-questionnaire, with respect to all measured outcomes. In both models, job satisfaction was the outcome best explained by psychosocial work stressors. This is interesting, as job satisfaction is not only the outcome related most closely to the self-rated work situation (43) but also a potential indicator for further factors such as organizational citizenship behavior (66, 67) and job performance (68). Furthermore, the COPSOQ model explained substantial proportions of variance in all outcomes, also in intention to leave the job and general health, compared to the ERI model. However, one has to consider that the COPSOQ encompasses more scales, which we assume to be the reason for its higher discriminant and predictive ability in our study. Even though the ERI-ratio was shown to be a potent predictor with respect to health behavior (69), endocrine, and immunological stress reactions (31, 70) and especially cardiovascular morbidity and mortality (71, 72), we could not predict strain outcomes using the ERI-ratio in the present sample. In contrast, the predictive power of the German COPSOQ regarding diagnosed clinical diseases and disorders is scarcely researched to date. A single ratio as provided by the ERI might be an easier and more precise measure for this purpose. However, it is not surprising that a shortened questionnaire, especially designed for large scale epidemiological studies, may not attain the same but nevertheless an adequate power in predicting the outcome of interest. Furthermore, our findings indicated the combination of both questionnaires not to be fruitful, as we could not determine any helpful contribution.

Looking at the results of the hierarchical regression analysis, it becomes apparent that, even when covariates had a high explanatory power, like age in general health (β = 0.408) or working volume in life satisfaction (β = 0.335), a reasonable impact of psychosocial working conditions on organizational and health-related outcomes can be observed in all models, strengthening the legitimacy and relevance of assessing psychosocial hazards in this industrial sector. Notably, work-privacy conflict was an important predictor of various strain indices. Issues of work–life balance are known to be of particular importance for health and well being (73–75). Even though efforts to implement measures that facilitate flexible work schedules have increased in the past years, conducting risk mitigation and workplace health promotion to comprehensively tackle work-privacy aspects, will surely result in enormous challenges for OHS as well as organizations, particularly with respect to blue-collar occupations organized in shift systems. Appropriate solutions might even demand new forms of work systems design. In this respect, we assume the most problematic aspects not to arise from designing measures for risk mitigation. As Nordlöf et al. (76) have demonstrated in a sample of the Swedish steel industry, a trade-off between productivity and safety in favor of the first seems to impede working safely. This was mainly determined by practical obstacles such as inappropriate or broke equipment, and the management’s expectations that production levels should be maintained constant even if staffing was low. Kiani and Khodabakhsh (77) found evidence for an association between employees’ perception of management safety practices and the tendency to report injuries in the Iranian steel industry. Finally, a poor access to mitigation resources has been shown to relate to the risk of workplace injury (78). This depicts the usual predominant role of the economic and technical element in such socio-technical systems. Keeping in mind that operational OHS, at least in Germany, has a consultative instead of an executive role, planning, designing, and implementing measures for risk mitigation might only play a minor role when confronted with daily business. We argue that psychosocial hazards have “hidden costs” which do not directly appear in organizations’ performance indicators, like for instance in sickness presentism. On the one hand, this is of course a matter of what is being measured in controlling departments. On the other hand, however, the interpretation of a latent psychosocial hazard—and this might apply for the construct of work stress in general—with a latent cost effect might seem more difficult for organizations than apparent and well-known business performance indicators. One can also speculate that data on health constraints, such as mental health, are mostly sourced from data of health insurance companies, and thus interpreted as being “something from the outside.” A possible solution in this respect might be to focus more on intervention studies. Improvements resulting from such measures can indicate the significance of psychosocial hazards in a sense of reversed causality. Conducting further research to clarify the hazardous character of psychosocial factors at work and their economic impact, and transferring this knowledge into standards, legal recommendations, and guidelines might be a practicable solution to support OHS in its ability to act.

### Strengths and Limitations of the Study

We are not aware of any other studies that quantitatively evaluated the role of psychosocial workload and related outcomes by validated instruments in the European steel industry. A straightforward comparison between COPSOQ and ERI appears to be difficult because of the different structural and theoretical assumptions of both instruments. As the present study is based on cross-sectional data, causality cannot be inferred. However, the theoretical framework proposes psychosocial workload to be the predictor and strain the criterion. Particularly in terms of occupational risk assessment, the hazardous character of psychosocial work-related stress is conceptualized as the mediator between risk exposure and health outcomes (79). A related issue in cross-sectional studies is the possibility of reverse and reciprocal causation (64). Referring to our analysis and to scientific literature, we claim that psychosocial factors at work have a plausible impact on associated outcomes. Yet, it has to be considered that for instance being highly content with work can likewise affect the perception of working conditions (80). Finally, the assessment of workload and strain *via* identical measures can result in common-method bias. However, by emphasizing the practical focus of this study, we assume a significant predictor to be relevant even if its variance might be inflated.

The final question that arises is how the present analyses generate new insights into occupational stress research and practice. We would like to stress that the broad and universal approach used in this study is only partially comparable with other investigations as such data from the European steel industry is hardly available. In contrast to most other studies, we included both blue- and white-collar workers to approximate the conditions of a heterogeneous workforce. Overall, it becomes apparent that the often so called “soft factors” seem to play an important role in an industrial environment that has mostly been researched in view of workers being exposed to “hard factors” in the past. Especially stressors relating to socioemotional aspects seem to have a reasonable impact. One can, therefore, conclude that the still more technically and medically oriented field of OHS should pay more attention to such factors. Notably, not all scales emerging as important predictors in the regression analyses revealed themselves as obvious risk factors in the comparison of average ratings with the reference values. There seems to be a need for statistical analysis in psychosocial risk assessment, which should be discussed further. Including confounders at the individual level, however, might compromise data protection. This implies that operational OHS in this sector will be confronted with new challenges, also in terms of risk mitigation and elimination, as interventions for mastering these risk factors might require comprehensive solutions at the organizational level. We are of course aware that the generalizability of the present results is limited, drawing conclusion with respect to a whole industry is, therefore, impossible. Rather than doing so, we want to provide a first insight into a multifaceted and economically important industrial sector, stressing the need for further investigations in this respect. Since especially psychosocial risk assessment proves to be a major challenge to organizations, we want to offer a perspective both for scientists and practitioners on how psychosocial hazards can be assessed in a complex working environment.

## Conclusion

Against the backdrop of psychosocial risk assessment, this study was conducted to gain insight into psychosocial stress and strain of employees working in the European steel industry. Considering the heterogeneous workforce of the steel industry, the German standard version of the COPSOQ- and the short version of the ERI-questionnaire were used to provide a broad measurement approach. Results revealed stronger effects sizes of partial eta^2^ within the COPSOQ scales in discriminating white- and blue-collar workers. Adjusted for covariates, the COPSOQ also showed a higher power in predicting outcomes like the intention to quit the job, job satisfaction, or personal burnout, presumably due to its greater variety of scales contrary to the ERI. The combination of both instruments did not result in any remarkable gain in explained variance. As the investigations revealed variables such as work-privacy conflict, meaning of work, and social support to be important predictors of the strain indices, our findings underline the significant role of especially socioemotional factors at work also in the European steel industry, indicating new fields of actions and challenges for OHS.

## Ethics Statement

This study was carried out in accordance with the recommendations and approved by the occupational review boards for data protection and work safety. No written informed consent was obtained; instead the common company specific procedures were applied: A working councils agreement including the data protection officers agreement ensuring compliance with all relevant privacy policy regulations according to German law was adopted. A member of the working council was present during the data collection process to ensure compliance with the agreement and to guarantee anonymity and voluntary participation.

## Author Contributions

All authors have made substantial contributions, including each of the following: (1) the conception and design of the study, or acquisition of data, or analysis and interpretation of data, (2) drafting the article or revising it critically for important intellectual content, or (3) final approval of the version to be submitted.

## Conflict of Interest Statement

The lead author discloses that he is partly employed in the company in which the study took place within his doctoral thesis. The authors wish to confirm that there are no further known conflicts of interest associated with this publication and there has been no significant financial support for this work that could have influenced its outcome.
